# Defining Dry Eye from a Clinical Perspective

**DOI:** 10.3390/ijms21239271

**Published:** 2020-12-04

**Authors:** Kazuo Tsubota, Stephen C. Pflugfelder, Zuguo Liu, Christophe Baudouin, Hyo Myung Kim, Elisabeth M. Messmer, Friedrich Kruse, Lingyi Liang, Jimena Tatiana Carreno-Galeano, Maurizio Rolando, Norihiko Yokoi, Shigeru Kinoshita, Reza Dana

**Affiliations:** 1 Department of Ophthalmology, Keio University School of Medicine, Tokyo 160-8582, Japan; 2 Department of Ophthalmology, Baylor College of Medicine, Houston, TX 77030, USA; stevenp@bcm.edu; 3 Eye Institute of Xiamen University, Xiamen 361102, China; zuguoliu@xmu.edu.cn; 4 Department of Ophthalmology, Quinze-Vingts Hospital, 75012 Paris, France; cbaudouin@15-20.fr; 5 Korea University Medical Center, Anam Hospital, Seoul 02841, Korea; hyomkim@hanmail.net; 6 Department of Ophthalmology, Ludwig Maximilian University, 80539 Munich, Germany; Elisabeth.Messmer@med.uni-muenchen.de; 7 Department of Ophthalmology, University of Erlangen-Nuremberg, 91054 Erlangen, Germany; Friedrich.Kruse@uk-erlangen.de; 8 Zhongshan Ophthalmic Center, Guangzhou 510060, China; lianglingyi@gzzoc.com; 9 Cornea & Refractive Surgery, Massachusetts Eye & Ear, Boston, MA 02114, USA; JimenaTatiana_CarrenoGaleano@MEEI.HARVARD.EDU (J.T.C.-G.); Reza_Dana@meei.harvard.edu (R.D.); 10 ISPRE OPHTHALMICS (Instituto di Medicina Oftalmica), 16129 Genoa, Italy; maurizio.rolando@gmail.com; 11 Department of Ophthalmology, Kyoto Prefectural University of Medicine, Kyoto 602-8566, Japan; nyokoi@koto.kpu-m.ac.jp; 12 Department of Frontier Medical Science and Technology for Ophthalmology, Kyoto Prefectural University of Medicine, Kyoto 602-8566, Japan; shigeruk@koto.kpu-m.ac.jp

**Keywords:** dry eye, definition, inflammation, tear film breakup, dry eye symptoms, dry eye signs, corneal epitheliopathy, neuropathic pain

## Abstract

Over the past decades, the number of patients with dry eye disease (DED) has increased dramatically. The incidence of DED is higher in Asia than in Europe and North America, suggesting the involvement of cultural or racial factors in DED etiology. Although many definitions of DED have been used, discrepancies exist between the various definitions of dry eye disease (DED) used across the globe. This article presents a clinical consensus on the definition of DED, as formulated in four meetings with global DED experts. The proposed new definition is as follows: “Dry eye is a multifactorial disease characterized by a persistently unstable and/or deficient tear film (TF) causing discomfort and/or visual impairment, accompanied by variable degrees of ocular surface epitheliopathy, inflammation and neurosensory abnormalities.” The key criteria for the diagnosis of DED are unstable TF, inflammation, ocular discomfort and visual impairment. This definition also recommends the assessment of ocular surface epitheliopathy and neurosensory abnormalities in each patient with suspected DED. It is easily applicable in clinical practice and should help practitioners diagnose DED consistently. This consensus definition of DED should also help to guide research and clinical trials that, to date, have been hampered by the lack of an established surrogate endpoint.

## 1. Introduction and Existing Definitions of Dry Eye Disease (DED)

Dry eye disease (DED) is a common ocular disorder affecting tens of millions of people; its incidence and prevalence in Asia is higher than in Europe and North America, suggesting that cultural or racial factors are involved in DED etiology [1,2,3,4,5,6].

The common perception of DED is one of a simple disease, primarily caused by decreased tear production—such as the aqueous tear deficiency (ATD) type of dry eye seen in Sjögren’s syndrome. However, evidence suggests that a number of conditions may contribute to an unstable tear film (TF) [7,8]. DED owing to decreased tear production and DED resulting from tear instability alone share some features but could also exist independently. Severe ATD also leads to a short tear breakup time (TBUT). 

The different clinical presentations of DED has led to some misunderstandings of the condition and the need for a consensus on a clinical definition. Over the years, various organizations have proposed different definitions and diagnostic criteria for DED.

In 2017, the most comprehensive of these was the Tear Film & Ocular Surface Society Dry Eye Workshop (TFOS DEWS II^®)^ Report, which was the result of a workshop involving DED specialists, academic researchers, optometrists and ophthalmologists [8]. The Asia Dry Eye Society also proposed a new definition in the same year [7], while the American Academy of Ophthalmology (AAO) proposed the new Dry Eye Syndrome Preferred Practice Patterns in 2018 [9]. All of these definitions emphasize that an unstable TF is a key feature of DED, but several discrepancies exist among the criteria.

Several of the commonly used DED definitions are summarized in Table 1. Different criteria/parameters have been used in each definition, some of which are difficult to measure clinically. Several definitions have also used instability of TF as a primary or secondary criterion for DED.

DED symptoms and short TBUT (≤5 s) are the criteria most often used to diagnose DED in three Asian countries (China, Japan and South Korea). Recent studies have emphasized the importance of an unstable TF, which can present as decreased TBUT in DED [18,19,20]. Consequently, in 2017, representatives from China, Japan and South Korea proposed the Asia Dry Eye Society definition of DED as “a multifactorial disease characterized by unstable TF causing a variety of symptoms and/or visual impairment, potentially accompanied by ocular surface damage” [7]. DED can be diagnosed by the presence of both decreased TBUT and subjective symptoms. However, ocular surface staining was not included as an essential diagnostic criterion in this iteration.

To achieve clinical consensus on the definition of DED, physician-scientists who specialized in DED met four times: in Chicago at the AAO Annual Meeting (2016), in New Orleans at the AAO Annual Meeting (2017), in Barcelona at the World Ophthalmology Congress (2018) and in Chicago at the AAO Annual Meeting (2018). The documents produced were approved by all participants, a list of which is provided at the end of this article (Appendix A).

### Clinical Implications

- The range of definitions has contributed to confusion and a lack of consistent diagnosis of DED across the globe.
- Tear film stability is a sensitive measure of tear dysfunction that can be easily measured, making it a clinically practical and reproducible marker of DED/tear dysfunction. It should be the key criterion in a clinical definition of DED.

## 2. A Proposed New Clinical Definition of Dry Eye Disease

Despite the various proposed definitions of DED, a single global consensus does not exist. Our discussion about a global definition started in 2016, and active discussions were held on four occasions based on the TFOS DEWS II^®^ Report, Asia Dry Eye Society, and the preferred practice patterns of AAO [7,8,9]. All discussions were conducted from a practical point of view. The primary question was how to make a proper diagnosis of DED in the clinic. The osmometer and noninvasive TBUT measurement were excluded, because these measurements are not available in all specialist eye clinics.

The final new definition proposed is: “Dry eye is a multifactorial disease characterized by a persistently unstable and/or deficient tear film causing discomfort and/or visual impairment, accompanied by variable degrees of ocular surface epitheliopathy, inflammation and neurosensory abnormalities.”

The definition emphasizes the importance of unstable TF, inflammation, discomfort and visual impairment. In addition, epitheliopathy and neurosensory abnormalities are included. According to the definition, a DED diagnosis can be made when patients have an unstable TF and ocular discomfort and or visual impairment. Since a patient with decreased TBUT and/or ocular discomfort and no ocular surface dye staining is easily misdiagnosed as having neurosis, TBUT should be measured using fluorescein dye for each patient with ocular discomfort. As we describe later, clinicians need to be cautious in order to not miss visual impairment due to DED, while ocular surface epitheliopathy, inflammation and neurosensory abnormalities should also be assessed in each patient. These important concepts will be discussed in detail in following sections.

### Clinical Implications

- The proposed new definition of DED is based on a consensus of agreement between DED experts in different regions worldwide.
- The new definition is aimed at providing practitioners with a practical tool that helps diagnose DED in the clinic.
- Using this simple clinical definition, all practitioners can make a precise and consistent diagnosis of DED.
- As the diagnosis can be made using the DED questionnaire and a slit lamp examination with fluorescein, all practitioners can easily apply this definition in their clinics following the recommendations outlined in this article.

## 3. Unstable Tear Film: The Importance of Tear Film Breakup Patterns

The current published data on tears, the TF and DED suggest that the primary mechanisms associating tear abnormalities to DED symptoms on opening the eye and, at the time of blinking, are tear film breakup (TFBU) and increased friction, respectively [21]. Ultimately, these mechanisms result in varying degrees of ocular surface epitheliopathy, inflammation and neurosensory response, each of which is responsible for specific DED symptoms [21,22].

### 3.1. Dynamic Process Involved in the Establishment of Tear Film to Understand Tear Film Breakup

Tear film dynamics comprise two processes, during which the precorneal TF is established. One process is the deposition of aqueous tears (ATs) to the corneal surface at eye opening [22,23,24,25,26,27], and the other is the redistribution of the TF after eye opening. During the deposition process, there are only polar lipids of the tear film lipid layer (TFLL), and in this process, polar lipids may contaminate the corneal surface when wettability is impaired. When the deposition process is terminated, the surface tension gradient of the TFLL causes the upward spread of the TFLL [22,23,24,25,27,28,29]. During this upward spread, a dimple (i.e., a transient thinning) is produced behind the leading edge of the spreading TFLL due to the upward drag of the aqueous TF exerted by the TFLL [28,30,31,32]. Thus, TF redistribution occurs, and when the upward spread of the TFLL and upward movement of the aqueous TF finishes, the redistribution is terminated. During the redistribution process, lower-tear meniscus-induced aqueous-TF thinning [33,34] occur simultaneously with the upward movement of the aqueous TF [22,24,25,27]. 

### 3.2. Tear Film Breakup Patterns for Tear Film-Oriented Diagnosis and Their Interpretations

A tear film-oriented diagnosis is a concept in which information is obtained on the integrity of the TFLL, the aqueous TF and the wettability of the corneal surface that is responsible for TFBU [24,25]. Although presently not a quantitative assessment, this information allows for (1) identification of the insufficient component of the TF or of the corneal surface epithelium responsible for TFBU, (2) subclassification of DED and (3) selection of optimal topical therapy. 

**Figure 1:**
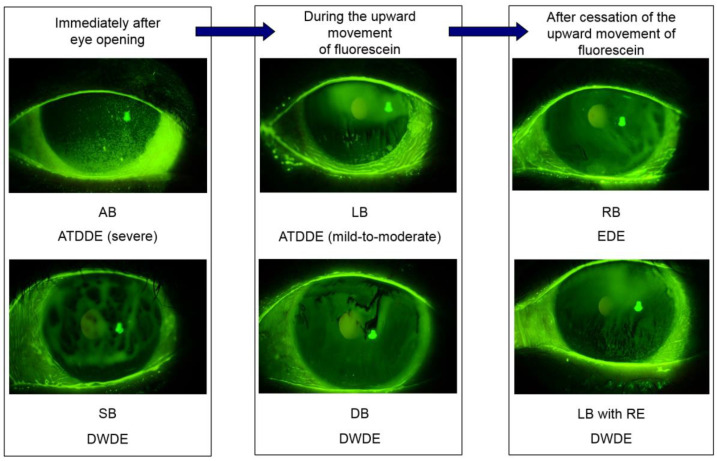
Fluorescein breakup (BU) patterns seen immediately after eye opening and after the eye is kept open. Related to the establishment of the tear film (TF) on the cornea, there are three phases for each fluorescein breakup pattern that can be seen. In the first phase, an area break (AB) or a spot break (SB), respectively, can be seen in cases of severe aqueous tear deficient dry eye (ATDDE) or decreased wettability dry eye (DWDE). In the second phase, a line break (LB) or a dimple break (DB), respectively, can be seen in cases with mild-to-moderate ATDDE or DWDE. In the third phase, a random break (RB) can be seen in cases of evaporative dry eye (EDE). RB is also seen in normal eyes, in which the BU time is within the normal range, i.e., generally, >10 s. In addition, a modification of BU patterns suggesting the association with DWDE is the rapid expansion of BU spots sometimes seen in LB and RB.

In severe ATD, not enough ATs are available for deposition. The result can be observed as either “no” or “incomplete” upward movement of fluorescein-stained aqueous TF or spread of the TFLL [27,35] (we termed this breakup (BU) pattern “area break” [22,24,25,27]; see Figure 1). However, even if ATs are sufficient, BU of TF can occur even when aqueous tears are sufficient due to a lack of mucins, which reduce the wettability of the epithelium (i.e., “spot break” (SB) [22,24,25,27] in Figure 1). In mild-to-moderate ATD, BU is likely to occur in the inferior part of the cornea (i.e., “line break” (LB) [22,24,25, in Figure 1). In addition, when the dimple encounters a corneal surface with decreased wettability, BU occurs (i.e., “dimple break” (DB) [22,25,27] in Figure 1). After the establishment of the TF, BU occurs even in normal eyes (i.e., “random break” (RB) [22,24,25,27] in Figure 1), and this happens more frequently in cases of DED with facilitated evaporation. The expansion of a tear film breakup becomes more rapid, depending on the decrease of wettability of the corneal surface [22,27,36,37,38], and it is possible that “line break” and “random break” can accompany the rapid expansion of the dark spot (i.e., “line break with rapid expansion” (LB with RE) in Figure 1).

### 3.3. Clinical Implications

- Tear-film oriented diagnosis is practical and clinically useful for clinicians engaged in the management of DED [22,24,27].
- Further advancement of this approach is expected through a deeper understanding of the function of each component of the ocular surface.
- TF stability is maintained by the components of the TF and ocular surface epithelium, and insufficiency of those components results in TFBU, a visible core manifestation of DED.
- Classification of TFBU patterns may elucidate the pathophysiology of DED.
- Clinicians can assess tear film-oriented diagnosis to learn more about the insufficient components responsible for TFBU, assess the specific DED subtype and, therefore, decide on the most appropriate treatment.

## 4. Ocular Discomfort: The Importance of Symptoms in Dry Eye Disease 

DED can be a frustrating disease for patients and ophthalmologists. Ocular discomfort is the most common complaint, and as a central feature of the disease, it is a fundamental component of the definition of DED [7,8,12]. In most cases, ocular discomfort is displayed before DED is diagnosed, and it is critical for monitoring the progression of the condition and response to treatments. 

Common ocular discomfort can be described as dryness, redness, foreign body sensation, heavy sensation, pain, light sensitivity, discharge, itching and eye fatigue. The type of ocular discomfort experienced by patients may be associated with particular DED subtypes, although there is considerable overlap between them. For example, in diurnal variation, DED symptoms tend to become more evident later in the day in patients with ATD dry eye [39]. In the classification proposed by the ADES, patients with short TBUT-type DED may experience more of the heavy sensation, and patients with staining-type DED may experience more of the foreign body-type sensation [7]. There is overlap among the symptoms of subtypes. 

### 4.1. Evaluation of Ocular Discomfort with Questionnaires

Ocular discomfort can be evaluated using patient-reported questionnaires (Table 2). Each questionnaire has advantages in certain scenarios and reflects the impact of DED on everyday function and health-related quality of life [8]. It is recommended that a validated symptom questionnaire be administered at first patient presentation. 

### 4.2. The Pathophysiology of Ocular Discomfort

In general, ocular discomfort is caused by an entity on the ocular surface activating the sensory nerve, and there are several reasons why this may occur. An unstable TF generates tear hyperosmolarity, inflammation and epithelial breakdown. This exposes and irritates nociceptive receptors on the ocular surface and leads to sensations of discomfort. Instability disturbs the optical property of the TF, becoming an additional source of discomfort. Sometimes, a patient may experience both problems. Mechanical abrasions from disorders of the lid margin, such as plugging of the meibomian gland orifice and conjunctivochalasis, can cause blink-related microtrauma to the ocular surface and affect tear dynamics. Insidious lid abnormalities, such as lagophthalmos, incomplete and decreased blinking or poor lid-to-globe apposition can prevent the formation of a stable TF and similar discomfort as DED. Inflammation is a result of a decreased or/and an unstable TF and epithelial defect and creates ocular irritation. Detailed note-taking to capture details of major symptoms and triggers helps to identify the origin of discomfort in DED. 

### 4.3. Ocular Discomfort and Incongruity with Signs

There are often discrepancies between signs and symptoms; thorough evaluation of both is critical in diagnosing DED. 

When patients demonstrate severe epithelial breakdown and inflammation but have no related discomfort; the neurotrophic status should be considered. Neurotrophic keratopathy in the context of dry eye disease usually stems from long-standing DED such as severe Sjögren’s syndrome and diffusive ocular surface keratinization in end-stage Stevens-Johnson syndrome, ocular cicatricial pemphigoid or chemical burn [51]. When patients report obvious discomfort without visible ocular surface signs, a component of neuropathic pain, also known as ocular neuralgia, is suspected. The typical complaint of neuropathic pain is hypersensitivity to light and wind, which is not relieved by topical anesthesia [52]. Depression and anxiety can cause chronic discomfort, which exceeds that predicted by clinical signs [53]. Patients with preclinical DED, such as long-term visual display terminal users or contact lens wearers, might also present with intermittent discomfort but very mild signs. In these cases, individuals may proceed to typical DED if risk factors are left uncorrected.

### 4.4. Clinical Implications

- Ocular discomfort is common and has a range of symptoms. A careful symptom consultation is recommended for diagnosis.
- Ocular discomfort should be evaluated with a variety of patient-reported questionnaires.
- A thorough evaluation of both signs and symptoms is critical for a correct diagnosis of DED and to determine treatment decisions.

## 5. Visual Impairment Caused by Unstable Tear Film

Patients with DED often have impaired visual function that affects functional activities and is caused by an unstable TF [54,55,56]. The TF is the first part of the eye traversed by light entering the eye, and a smooth stable TF is essential for good visual function [57,58,59]. A recent study showed that an unstable TF causes increased accommodative microfluctuations, placing a great burden on the ciliary muscle [60,61]. Once the TF has been stabilized, the symptoms are resolved when the fluctuations stop. 

In the context of DED, functional visual acuity assesses the visual functions related to the unstable TF [58,62]. As compared with the normal visual acuity measurement, functional visual acuity provides a measure of the real-life functional ability of visual perception. Impaired functional visual acuity can be recovered by treating DED with punctal occlusion [63] or diquafosol sodium [64,65,66,67], which stabilize the TF. Blue light, often used in computer and smartphone screens, has a short wavelength, which is easily scattered by an uneven surface, i.e., an unstable TF. Thus, DED patients have more difficulty seeing blue light, and the light environment can be improved by removing blue light components [68]. The recent acceptance of blue light-reducing glasses for computer users should help with clinical or preclinical DED.

### Clinical Implications

- Given that DED can impair visual function, practitioners should properly evaluate DED symptoms and visual impairment.
- As simple visual acuity measurements cannot detect visual impairment associated with DED, it is possible that practitioners underestimate the extent of visual impairment.
- Assessments such as functional visual acuity can be used to assess visual function related to unstable TF.
- Simple measures such as reducing blue light emitted by devices can be used to improve visual function in those with unstable TF.

## 6. Epitheliopathy

Epitheliopathy commonly occurs in DED and contributes to the instability of TF. The distribution and severity of corneal disease and conjunctival epithelial disease differ among aqueous tear deficient and sufficient dry eye/tear dysfunction conditions and may not be clinically detectable [55,69,70,71].

### 6.1. Corneal Disease 

Corneal epithelial disease is more prevalent and severe in ATD syndromes, particularly in Sjögren’s syndrome and inflammatory conditions such as Stevens–Johnson syndrome and graft versus host disease, than aqueous tear sufficient dry eye [55,71]. Fluorescein is most frequently used to identify and measure the severity of corneal epithelial disease [72], and several severity grading schemes have been proposed [71,73]. Staining develops due to corneal epithelial death, dysfunction or an altered permeability barrier that has been observed in DED patients and experimental mouse models [72,74,75,76,77,78]. Corneal epithelial disease may also be detected by imaging techniques. 

Increased levels and activity of matrix metalloproteinases (MMPs) such as MMP-9 and its activator MMP-3 contribute to corneal barrier disruption [79,80]. MMP-9 knockout mice are resistant to corneal barrier disruption when exposed to desiccating stress, and MMP inhibitors such as corticosteroids and doxycycline have shown potential in preventing desiccation-induced corneal epithelial barrier disruption in animal models [81]. This can lead to accelerated desquamation of the apical epithelium and, in some cases, full thickness epithelial sloughing, which can predispose one to sight-threatening stromal ulceration and opacification. Mice subjected to desiccating stress show increased expression of cornified envelope precursors in the corneal epithelium, and patients with Stevens–Johnson syndrome can show cornification of the corneal epithelium [80,82,83].

### 6.2. Conjunctival Disease

The conjunctiva has a stratified columnar epithelium with goblet cells that produce tear-stabilizing gel-forming mucins and immunomodulatory factors such as retinoic acid and transforming growth factor (TGF)-β2 [84,85,86]. Desiccation and lacrimal hyposecretion with a loss of tear trophic factors, activation of stress kinases and inflammatory cytokines, such as interferon gamma (IFN-γ), alter conjunctival differentiation [82,87]. The Th2 cytokine interleukin (IL)-13 stimulates proliferation and mucus production, while the Th1 cytokine IFN-γ induces goblet cell entrapment, the expression of cornified envelope precursors, decreased mucus production, unresponsiveness to cholinergic stimulation, endoplasmic reticulum stress and unfolded protein response and apoptosis [88,89,90,91,92,93,94,95]. Conjunctival disease is detected by vital dye staining with lissamine green or Rose Bengal dyes.

Goblet cell loss in the conjunctival epithelium is a well-recognized feature of ATD [96,97,98,99,100,101,102]. The most severe ocular surface diseases, such as Stevens–Johnson syndrome, mucous membrane pemphigoid, graft versus host disease and severe alkali burns involving the conjunctiva, often have a complete loss of conjunctival goblet cells [69,103,104,105]. Goblet cell loss is accompanied by decreased concentration of the goblet cell mucin, MUC5AC, in tears [106] and correlates with increased conjunctival lissamine green and Rose Bengal dye staining and rapid TBUT [70,107,108,109]. Eyes with extensive goblet cell loss often have instantaneous TBU.

### 6.3. Clinical Implications

- Epitheliopathy is frequently observed in DED but may be clinically undetectable in some patients.
- Epitheliopathy can contribute to instability of TF, blurred vision, altered permeability, nociceptor exposure and sensitization and pain in DED.

## 7. Dry Eye Disease and Inflammation 

The surface epithelium and the TF contain dendritic cells, macrophages, mast cells, lymphocytes, fibroblasts, cytokines and additional immune mediators, which guard against pathogens and other environmental threats. DED may develop when this homeostasis is disrupted by aqueous tear deficiency, TF hyperosmolarity and inflammation of the ocular surface microenvironment [84]. Local, autoimmune-mediated ocular surface dysfunction may also play a role in DED pathogenesis [70,87].

Other factors that may contribute to inflammation of the ocular surface include: microtrauma; hyperosmotic stress; aging; irritants such as microbial antigens and UV light and infection and systematic inflammatory diseases, conditions and disorders (i.e., auto- and allo-immune diseases, neuroinflammation and sterile inflammation). In early phase inflammatory DED, any of these factors can stimulate the production of inflammatory cytokines and their subsequent immune responses. 

### 7.1. Innate Immune Responses in Dry Eye Disease

**Figure 2:**
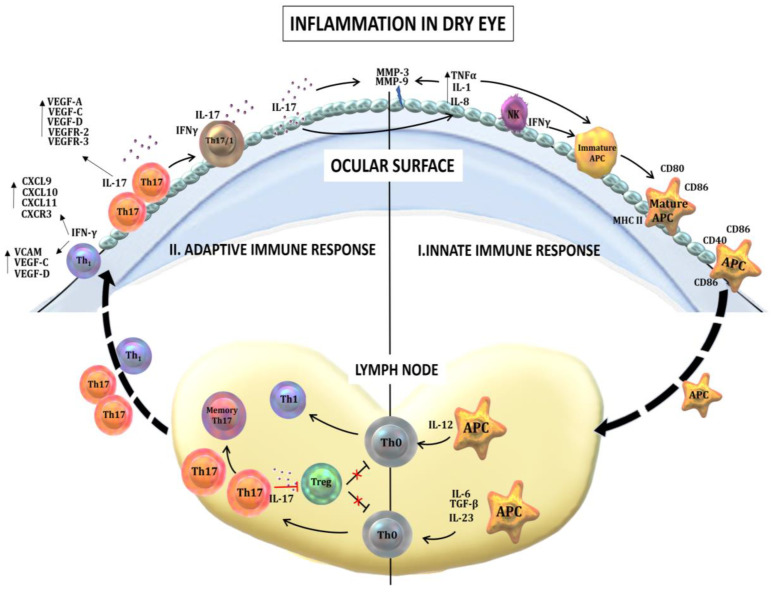
Inflammation in dry eye disease (DED). (I) The innate immune response in DED is mediated by multiple cells. Natural killer (NK) cells secrete interferon gamma (IFN-γ), which, along with other proinflammatory cytokines upregulated at the ocular surface, such as interleukin (IL)-1, IL-6 and tumor necrosis factor alpha (TNF-α, promote the maturation of antigen-presenting cells (APCs) through inducing the upregulated expression of major histocompatibility complex (MHC)-II, CD40, CD80 and CD86. In addition, these proinflammatory cytokines lead to the production of matrix metalloproteinases (MMPs) in epithelial cells, which further abrogate the corneal epithelial barrier. (II) The adaptive immune response in DED is initiated by the activation and migration of activated APCs toward the regional draining lymph nodes, where they prime naïve T cells (Th0) to differentiate and expand into IL-17-secreting T-helper cell 17 (Th17) cells and IFN-γ-secreting Th1 cells. IL-17 inhibits the regulatory T cells (Treg) suppressive function. Effector T cells migrate to the ocular surface, where they secrete an array of effector cytokines. Th1 cells secrete IFN-γ, which leads to the upregulated production of chemokines such as IL-9 (CXCL9), CXCL10, CXCL11, CXCR3 and cell adhesion molecules (CAMs), facilitating the ingress of more inflammatory cells to the ocular surface. IL-17 stimulates the production MMP-3 and MMP-9, leading to epithelial cell damage. IL-17 also increases the expression of vascular endothelial growth factor (VEGF)-A, VEGF-C, VEGF-D, VEGFR-2 and VEGFR-3. With the resolution of ocular inflammation, a small portion of Th17 cells convert into long-lived memory Th17 cells mediating chronic inflammation in DED.

The immunopathogenic mechanisms in DED are complex and are summarized below and in Figure 2. In DED, multiple cell types of the innate immune system mediate inflammatory damage to the ocular surface. Innate immune responses include the upregulation of Toll-like receptors (TLRs) and nucleotide-binding domain, leucine-rich containing family and pyrin domain containing-3 (NLRP3) [110,111]. TLR signaling pathways stimulate the production of transcription factors, including activator protein (AP)-1, nuclear factor kappa-light-chain-enhancer of activated B cells (NF-κB) and interferon regulatory factor (IRF)-5 [112].

The conjunctival epithelium and tears of patients with DED also contain elevated levels of inflammatory mediators such as ILs-6 and -23, tumor necrosis factor (TNF)-α and TGF-β1. These inflammatory mediators promote differentiation of CD4+ T-helper cell 17 (Th17), which secretes IL-17 cytokines (IL-17A and IL-17F), the major pathogenic cells promoting DED (see below), which will be described in the next section. Other proinflammatory cytokines produced by epithelial cells and macrophages participating in the innate immune response include IL-1α, IL-1β, IL-2, IL-5 and chemokines such as IL-8 (CXCL8) [113]. 

TNF-α and IL-1β promote the expression of intercellular adhesion molecule 1 (ICAM-1), as well as the expression of major histocompatibility complex (MHC) class II and co-stimulator B7 (CD80 and CD86) on antigen-presenting cells (APCs), which mediate APC-T-cell interactions. IL-1β stimulates the production of TNF-α and MMP-3 [95,114,115,116]. IL-1β and IFN-γ are associated with squamous metaplasia of epithelial cells [87,117]. IFN-γ decreases goblet cell differentiation and mucus production. In vitro, corneal epithelial cells express MHC class II with the stimulation of IFN-γ. Conjunctival epithelia overexpress MHC class II in a cytokine-rich environment, with a decrease of goblet cells. Some studies suggest that goblet cells play a critical role in maintaining ocular surface immune tolerance [85], and their disruption leads to dendritic cell maturation [118]. Fas and the Fas ligand are also elevated in DED [119,120,121] and regulate the apoptosis of ocular surface cells in the extrinsic pathway [122].

### 7.2. Adaptive Immune Responses in Dry Eye Disease

T cell infiltration in the ocular surface is a hallmark of chronic inflammation, and multiple studies show that autoreactive T cells play a central role in DED [91,121,123]. APCs activated by inflammatory mediators migrate to local lymph nodes, where they prime naïve T cells to polarize into effector CD4+ T-helper (Th1) and Th17 cells [123,124]. These effector cells then migrate to the conjunctiva, secreting IFN-γ and IL-17, and upregulate the production of a variety of chemokines [113,125,126] and cell adhesion molecules (CAMs), as well as vascular endothelial growth factor (VEGF)-C and VEGF-D. 

Th17 cell differentiation is promoted by the transcription factor RORγt, which is expressed in response to IL-6, TGF-β and IL-23 [127]. Th17 cells secrete IL-17 [128,129], which increases proinflammatory cytokines and MMP3/MMP9 expression in response to desiccating stress. IL-17-secreted by Th17 cells also increase the expression of VEGF-C and VEGF-D, and their receptors VEGFR-2 and VEGFR-3, promoting corneal lymphangiogenesis [113] without associated hemangiogenesis [130,131]. In DED, Th17 homing is mediated by CCL20 [128]. In addition, a unique Th17 population, secreting both IL-17 and IFN-γ (Th17/1), was identified that amplifies ocular surface inflammation in DED, and the generation of Th17/1 is facilitated by environmental IL-12 and IL-23 [132]. Furthermore, chronic inflammation in DED is mediated in the long term principally by long-lived Th17 effector cells, namely memory Th17 cells [133]. These pathogenic memory Th17 cells are maintained by environmental IL-7 and IL-15 [134]. When IL-17 is blocked, DED severity significantly decreases. 

The regulatory T cell (Treg; CD4+CD25+Foxp3+) attenuates the immune response by reining in the autoreactive T cells. Treg expresses integrin αEβ7 (CD103), which limits naïve T cell priming via the secretion of the anti-inflammatory cytokines (TGF-β and IL-10) and Treg/APC or Treg/T effector cell interactions [135]. There are two subsets of Tregs: one originating from the thymus (natural Treg, nTreg) and the other induced in the periphery in the presence of IL-2 and TGF-β (induced Treg, iTreg). Inadequate suppression by Tregs leads to disruption of immune tolerance and the generation of effector T cells. The Th17 cells in DED were found to be resistant and functionally antagonistic to Treg activity [123]. The dysfunction of Treg suppression exacerbates ocular surface inflammation in models of DED [123].

### 7.3. Neurogenic Inflammation

Mechanical and inflammatory damage to the ocular surface may lead to the synthesis of neurotrophic factors that stimulate nerve growth but can also alter the corneal nerve structure, as seen in the sub-basal corneal nerve plexus, and promote inflammation, which can lead to nerve degeneration. Epithelial damage induced by hyperosmolarity stimulates the release of vasoactive neuropeptides and the activation of immune cells, resulting in plasma extravasation and local inflammation [136].

Neuropeptides and neurotrophins found at the ocular surface have also been associated with nonspecific corneal pain and other DED symptoms. 

These neuropeptides maintain neurogenic inflammation and work together with the immune responses, not only protecting the ocular surface but, also, initiating the immune response in DED and regulating its chronicity. Thus, nerve-derived factors can both attenuate and amplify DED severity depending on the context in which they function. 

Hormonal variations may also affect the ocular surface in DED. Studies in animals have reported that estrogen may upregulate MMP-2 and MMP-9 and increase the expression of inflammatory genes such as IL-1β, IL-6 and IL-8 in human corneal epithelial cells. By contrast, androgens may reduce macrophage TNF-α and IL-1β expression. However, some studies have found that estrogen also may decrease DED severity [137].

In summary, there is increasing evidence that a wide array of immune and nerve-derived factors can influence one another through complex mechanisms that result in damage to the ocular surface epithelium, changes in the sub-basal nerve plexus nerve density and function and development of DED symptoms. Much work remains to be done to better elucidate the interaction of these factors in chronic DED.

### 7.4. Clinical Implications

- Inflammation is a key aspect of DED pathogenesis and severity.
- Methods for identification of DED include the RPS InflammaDry Detector, an immunoassay test for the detection of elevated levels of the MMP-9.
- The control of inflammation, chronically or during clinical flares, is necessary for managing patient discomfort and ocular surface disease.
- While there can be a lack of correlation between patient symptoms and signs of ocular surface disease, managing inflammation can help attenuate both the signs and symptoms of DED.

## 8. Neurosensory Abnormalities

In its new definition of DED [8], DEWS II introduced the concept of neurosensory abnormalities, emphasizing the central role of the corneal nerves and the neurosensory loop driven by the trigeminal pathway. The role played by corneal innervation has extensive consequences in terms of neurotrophic balance, the regulation of tear secretion and ocular surface inflammation, with the emerging concepts of neuroimmune synapse or neurogenic inflammation illustrating the interactions between the immune system and the trigeminal pathway.

The human cornea is densely innervated by the sensory fibers of the trigeminal nerve, with relatively few nerve bundles penetrating the cornea, but, also, a very dense network of intraepithelial nerve endings with a variety of mechanical, chemical and thermal receptors [52]. Corneal nerves also play a major role in controlling lacrimal gland secretion, thus regulating tear volume and composition through the trigeminal–facial nerve loop. 

Neurosensory abnormalities in the ocular surface may participate in three different but closely related mechanisms in DED, which may act as initiators or consequences of the disease—namely, neurogenic inflammation, neuropathic pain and neurotrophic keratopathy. 

Neurogenic inflammation is a key factor of the vicious cycle involved in DED [138]; neuropathic pain and neurotrophic keratopathy may explain the frequent discrepancies between DED signs and symptoms, with corneal hypoesthesia leading to the paradoxical condition of a highly damaged cornea with few or no symptoms, whereas neuropathic pain corresponds to highly symptomatic patients despite no, or barely detectable, corneal damage.

As a causative role in DED, alterations of the corneal innervation loop are primary mechanisms of a wide variety of conditions—among them, corneal refractive surgery, where DED has become the most frequent and disabling complication. Most causes are iatrogenic. However, structural and functional alterations of the sub-basal corneal nerves were identified in other types of DED, with reduced nerve density and branching, increased tortuosity, bead-like patterns, greater nerve width and aspects of neuromas, a pattern that would appear more closely associated with neuropathic pain [139,140]. The reduction in nerve density was highly correlated with an increased density of inflammatory cells and the ocular surface disease index, illustrating the close relationships between nerves, symptoms and corneal inflammation [141].

A key role of sustained neural stimulation in response to increased osmolarity, epithelial alterations and mechanical stress is the release of proinflammatory neuropeptides by the nerve endings, leading to neurogenic inflammation, a prominent mechanism of DED. 

However, some patients do not show significant tissue damage that would explain their high levels of symptoms. This raises the concept of neuropathic pain, where the symptoms may resemble those of DED, and the signs are mostly limited or only associated with mild tear abnormalities or subclinical changes or are only detectable using confocal microscopy or impression cytology through the expression of tear or conjunctival biomarkers of inflammation [140,142,143]. DED and neuropathic pain often have similarities in their development [144], but the continuous firing of corneal nerve receptors may result in a lowered stimulatory threshold. This is typically experienced as persistent irritation or pain in response to a normally innocuous stimulation—namely, allodynia and/or increased impulse transmission following suprathreshold stimulation, with abnormally increased pain sensation—namely, hyperalgesia [52].

When stimulation persists, patients may progressively develop mechanisms of central neuronal sensitization within the trigeminal complex in the brainstem and, in higher-order neurons in the brain, leading to neuropathic pain symptoms [52,145,146]. Chronic neuropathic pain persists irrespective of the ocular surface findings and initial mechanisms, thus resisting etiologic or symptomatic treatments [52,145]. Indeed, a significant part of these cases are associated with depression or anxiety and may also involve patients with a general pain syndrome. Small fiber neuropathy has been frequently associated with Sjögren’s syndrome, and a patient’s general condition should be addressed for holistic patient care [147].

Conversely, an opposite condition may be observed in DED when chronic damage to corneal nerves may cause decreased sensitivity of the cornea, with low levels of symptoms despite a highly damaged ocular surface. This might explain one aspect of the well-known sign/symptom discrepancy in DED, but, additionally, the neurotrophic consequences of nerve abnormalities may participate in highly aggravating corneal damage. Indeed, even if nerve transection is mostly transient in DED induced by refractive surgery, sustained anatomical or functional nerve dysfunction deprive the cornea of neurotrophic factors and prevent the corneal epithelium from recovering and, in the most severe cases, leading to a neurotrophic keratopathy. Decreased corneal sensitivity has been observed in patients with high variability, but there is a significant correlation with disease severity, indicating progressive corneal nerve damage and/or dysfunction in a chronically damaged ocular surface [148].

Three stages of neurotrophic keratopathy have been described [149], and the features observed in stage 1 are very similar to those of severe DED. Patients may present with painless sensations or few symptoms, despite damaged corneas. However, symptoms driven by conjunctival inflammation or hyperactive nerve fibers may mask localized areas where the cornea is anesthetized, which may lead to the delay or underestimation of the neuropathic mechanisms. In this case, inappropriate treatment can lead to more severe damage and the involvement of deeper layers of the cornea. Therefore, an assessment of corneal sensitivity is required for weakly symptomatic patients with corneal epitheliopathy or epithelial damage resisting adapted therapies [150].

From the clinical point of view, neurosensory abnormalities play multiple roles in DED, as the primary source of disabling symptoms and sensations, as well as by disrupting the homeostasis of the ocular surface, blocking the secretion of neurotrophic factors or stimulating chronic inflammation. Their role, therefore, deserves much attention, and developments of new therapies can benefit from recognition of this core mechanism and from further research.

### Clinical Implications

- The human cornea is densely innervated and corneal nerves participate in the stability of the ocular surface. Their continuous stimulation by a disrupted TF may cause symptoms.
- Neurosensory abnormalities may participate in three different but closely related mechanisms in DED: neurogenic inflammation, neuropathic pain and neurotrophic keratopathy. These may contribute to the lack of correlation often observed between DED signs and symptoms.
- Neurosensory abnormalities in DED must be studied further but should be considered when assessing signs, symptoms and treatment responsiveness in the clinic.

## 9. Implications for Regulatory Science

Novel treatment pathways for DED are being explored with innovative pharmaceutical agents. Initial studies at the basic research level are followed by translational research, aimed at investigating efficacy using in vitro and animal experiments. At this stage, it is vital to determine if a newly discovered agent or molecule has been accepted for medical use in other fields by government regulatory agencies, so that the requirements for nonclinical studies, including toxicology studies, may not be necessary.

There are important issues to be addressed when considering the introduction of medical products for the treatment of DED, including that only a few products specifically aimed at the treatment of DED have been officially approved by government regulatory agencies. It is likely the result of conceptual discrepancies between academic science, and regulatory science could account for the small number of products approved by regulatory agencies for DED. In many countries, approved medical products are categorized into three major groups: pharmaceutical agents (drugs), medical devices and products for regenerative medicine. Of those, most medical products designed for DED treatment fall into the categories of pharmaceutical agents and medical devices and, as such, are strictly regulated by the laws and/or guidelines set by each government.

Academic research scientists tend to focus on efficacy, while regulatory agencies are primarily concerned with safety, which is a vital perspective of regulatory science. It is always possible that patients will unexpectedly have adverse events caused by pharmaceutical agents and/or contaminated pathologic microorganisms and viruses, illustrating why strict government regulation is important. In order to assess the safety and efficacy in clinical trials, the primary endpoint should be set not only from the perspective of clinicians and researchers but should also seek to assess the restoration of visual function. 

In the field of DED research, one of the difficulties is the absence of global consensus between regulatory agencies on the definitions of TF abnormalities, including the cut-off value for TBUT and the Schirmer 1 test. Each country has established its own cut-off value (Table 3). Moreover, regulations for the approval of DED products in clinical trials vary between the US, the EU and Japan (Table 4) [151,152,153,154]. Another difficulty is the lack of an established surrogate endpoint for global clinical trials.

Several important points differ slightly between the processes of research and development and those of product development, especially on the equal quality of products. These points might be essential for future internationally harmonized clinical trials for DED products—again, illustrating the importance of regulatory science with its requirement for an accumulation of well-controlled evidence-based data.

In summary, the safety profile of study drugs should be the object of thorough attention. Real-world data analysis could be useful in such circumstances, following government approval of pharmaceutical agents, and a rational use of medicine has also been proposed [155,156].

### Clinical Implications

- Academic science tends to focus on efficacy; however, safety—a critical consideration for drug research—is key for regulatory agencies.
- The establishment of a well-organized surrogate endpoint is important for worldwide clinical research, as currently there is no global consensus on the measure of TF abnormalities in clinical trials.

## 10. Conclusions

Many common diseases, including ocular diseases, are caused by the interaction of human genes and the environment. The numbers of DED patients have increased dramatically in the past decades (up to 30–40% in some populations) [2,3,4,5,6], likely to be predominantly caused by environmental changes. The increase in visual tasks, an aging society, sleep deprivation, impairments in circadian rhythms, lack of exercise, increase in obesity and sedentary lifestyles could also contribute to the increase in DED incidence [157,158,159,160,161]. In one sense, DED can be considered a lifestyle disease. Although we have emphasized the importance of TFBU, the control of inflammation is also important by employing various therapeutics [84,162,163,164]. Since the consensus of the National Eye Institute/Industry Workshop on Clinical Trials in Dry Eye in 1995 [10], the definition and diagnostic criteria have been changing partially to reflect the changing nature of DED and the causes of DED, in our modern, visually oriented society where the prolonged use of computers or smartphones is common in daily life. The activities of society will continue to evolve over the next generations and will affect the characteristics of DED in the future.

The purpose of this paper was to reach a global consensus among ophthalmologists who examine DED patients almost every day. There have been alternative definitions of DED in the past, but here, we proposed a simple definition, with emphasis on the importance of TBU and subjective symptoms or visual impairment. As stated in Section 2, the new definition proposed is: “Dry eye is a multifactorial disease characterized by a persistently unstable and/or deficient tear film causing discomfort and/or visual impairment, accompanied by variable degrees of ocular surface epitheliopathy, inflammation and neurosensory abnormalities”. We hope this consensus will contribute to the diagnosis of DED in clinics and aid progress in the DED research field.

## Figures and Tables

**Table 1:** Dry eye disease definitions proposed from 1995 onwards.

Group	Instability of Tear Film	Global Consensus
NEI/Industry Workshop on Clinical Trials in Dry Eye (1995) [10]	−	−
Pflugfelder (2003) [11]	P	−
TFOS DEWS I (2007) [12]	S	+
TFOS DEWS II (2017) [8]	S	+
AAO PPP (2018) [9]	P	−
Japanese Dry Eye Research Group (2006) [13]	−	−
Korean Corneal Disease Study Group [14]	P	−
Chinese Medical Association Ophthalmic Branch Corneal Group [15,16]	P	−
Asia Dry Eye Society 2017 [7]	P	+
Japanese Dry Eye Research Group (2016) [17]	P	−
New Global Consensus Definition	P	−

P and S indicate if tear instability was a primary (P) or secondary (S) criterion in the definition, respectively. + and − in the Global Consensus column indicate if the definition was approved by consensus of an international group or organization. NEI: National Eye Institute; TFOS: Tear Film & Ocular Surface Society; DEWS: International Dry Eye Workshop; AAO PPP: American Academy of Ophthalmology Preferred Practice Patterns.

**Table 2:** Questionnaires used to evaluate symptoms of dry eye disease (DED).

Questionnaire	Initial Launch	No. of Questions	Screening Criteria for DED	Recommendations for Use
McMonnies [40]	1987	12	>14.5	
Ocular Surface Disease Index (OSDI) [41]	2000	12	Mild: 13–22 Moderate: 23–32 Severe: ≥33	Suitable for research and ATD
National Eye Institute’s Visual Function Questionnaire (NEI-VFQ 25) [42]	2001	25		Adapted for moderate-to-severe dry eye Strongly correlated with OSDI
Dry Eye Questionnaire (DEQ) [43]	2002	23		
Ocular Comfort Index (OCI) [44]	2007	12		The only questionnaire that provides valid measurement based on Rasch analysis
Symptom Assessment in Dry Eye (SANDE) [45]	2009	2		Suitable for clinical assessment
Standard Patient Evaluation of Eye Dryness (SPEED) [46]	2009	12	No symptoms: 0 Mild-to-moderate: 1–9 Severe: ≥10	Suitable for Meibomian gland dysfunction
Impact of Dry Eye on Everyday Living (IDEEL) [47]	2011	57	Mild 40–50 Moderate 51–63 Severe > 64	
Dry Eye-related Quality-of-life Score (DEQS) [48]	2013	15		
5-Item Dry Eye Questionnaire (DEQ-5) [49]	2013	5	keratoconjunctivitis sicca: > 6 Suspected Sjögren’s syndrome: >12	
Chinese Dry Eye Questionnaire [50]	2015	12		Questionnaire validated in the Chinese population

ATD = aqueous tear deficiency.

**Table 3:** Abnormal values of the tear breakup time and Schirmer I test in each country.

Location	Tear Breakup Time (Seconds)	Schirmer I Test (mm)
USA	Not determined	Not determined
Germany	Abnormal < 10 Severe abnormal < 5	Abnormal < 10 Severe abnormal < 5
France	≤5	≤5
Italy	≤7	≤5
Japan	≤5	≤5
China	5 < Dry eye disease suspected ≤ 10 Dry eye ≤ 5	5 < Dry eye disease suspected ≤ 10 Dry eye ≤ 5
South Korea	5 < Dry eye suspected ≤ 10 Dry eye ≤ 5	5 < Dry eye suspected ≤ 10 Dry eye ≤ 5

**Table 4:** Current approval requirements in the US, EU and Japan.

Location	Sign	Symptom	Duration of Clinical Study (Months)
US	Primary Any *	Primary Any *	3
EU	Primary Any *	Primary Any *	6
Japan	Primary Staining	Secondary Any *	1

* These are not specified in the US and EU; however, they must be validated for the treatment of DED.

## Abbreviations

AAO	American Academy of Ophthalmology
AP	activator protein
APC	antigen-presenting cell
AT	aqueous tear
ATD	aqueous tear deficiency
ATDDE	aqueous tear deficient dry eye
BU	breakup
CAM	cell adhesion molecules
DB	dimple break
DED	dry eye disease
DWDE	decreased wettability dry eye
EDE	evaporative dry eye
ICAM-1	intercellular adhesion molecule
IFN	interferon
IL	interleukin
LB	line break
MGD	meibomian gland dysfunction
MHC	major histocompatibility complex
MMP	matrix metalloproteinase
NF-κ B	nuclear factor kappa-light chain-enhancer of activated B cells
NLRP3	nucleotide-binding domain, leucine-rich containing family, pyrin domain containing-3
RB	random break
RE	rapid expression
SB	spot break
TBU	tear breakup
TBUT	tear breakup time
TF	tear film
TFBU	tear film breakup
TFLL	tear film lipid layer
TGF	transforming growth factor
TLR	Toll-like receptor
TNF	tumor necrosis factor
Treg	regulatory T cell
VEGF	vascular endothelial growth factor

## Appendix A: Participant List

Christophe Baudouin, Quinze-Vingts Hospital, Paris, France
Reza Dana, Massachusetts Eye & Ear, Boston, MA, USA
Hyo Myung Kim, Korea University Medical Center, Anam Hospital, Seoul, South Korea
Shigeru Kinoshita, Kyoto Prefectural University of Medicine, Kyoto, Japan
Friedrich Kruse, University of Erlangen-Nuremberg, Erlangen, Germany
Zuguo Liu, Eye Institute of Xiamen University, Xiamen, China
Elisabeth M. Messmer, Ludwig Maximilian University, Munich, Germany
Stephen C. Pflugfelder, Baylor College of Medicine, Houston, TX, USA
Maurizio Rolando, University of Genoa, Genoa, Italy
Kazuo Tsubota, Keio University School of Medicine, Tokyo, Japan
Norihiko Yokoi, Kyoto Prefectural University of Medicine, Kyoto, Japan
